# The HIRA complex that deposits the histone H3.3 is conserved in *Arabidopsis* and facilitates transcriptional dynamics

**DOI:** 10.1242/bio.20148680

**Published:** 2014-08-01

**Authors:** Xin Nie, Haifeng Wang, Jing Li, Sarah Holec, Frédéric Berger

**Affiliations:** 1 Temasek Lifesciences Laboratory, 1 Research Link, National University of Singapore, 117604 Singapore; 2 Department of Biological Sciences, National University of Singapore, 14 Science Drive 4, 117543 Singapore

**Keywords:** *Arabidopsis*, Chromatin, Histone variant, Histone modification, Differentiation

## Abstract

In animals, replication-independent incorporation of nucleosomes containing the histone variant H3.3 enables global reprogramming of histone modifications and transcriptional profiles. H3.3 enrichment over gene bodies correlates with gene transcription in animals and plants. In animals, H3.3 is deposited into chromatin by specific protein complexes, including the HIRA complex. H3.3 variants evolved independently and acquired similar properties in animals and plants, questioning how the H3.3 deposition machinery evolved in plants and what are its biological functions. We performed phylogenetic analyses in the plant kingdom and identified in *Arabidopsis* all orthologs of human genes encoding members of the HIRA complex. Genetic analyses, biochemical data and protein localisation suggest that these proteins form a complex able to interact with H3.3 in *Arabidopsis* in a manner similar to that described in mammals. In contrast to animals, where HIRA is required for fertilization and early development, loss of function of HIRA in *Arabidopsis* causes mild phenotypes in the adult plant and does not perturb sexual reproduction and embryogenesis. Rather, HIRA function is required for transcriptional reprogramming during dedifferentiation of plant cells that precedes vegetative propagation and for the appropriate transcription of genes responsive to biotic and abiotic factors. We conclude that the molecular function of the HIRA complex is conserved between plants and animals. Yet plants diversified HIRA functions to enable asexual reproduction and responsiveness to the environment in response to the plant sessile lifestyle.

## INTRODUCTION

In flowering plants, a series of specific phase transitions control several aspects of the sporophytic life, including embryo maturation, seed germination, and the initiation of flowering. The switch between transcriptional activities of large groups of genes during major developmental transitions is accompanied by dynamic changes of chromatin marks including histone 3 lysine 27 di/tri-methylation (H3K27me2/3) deposited by the Polycomb repressor complex 2 (PRC2) (Holec and Berger, 2012; Gentry and Hennig, 2014). The impact of PRC2 on developmental transitions is likely very ancient as PRC2 activity is conserved in the moss *Physcomitrella patens* and regulates the transition between the haploid gametophyte and the diploid sporophyte (Mosquna et al., 2009; Okano et al., 2009).

The dynamics of chromatin states required to accompany the transition from one developmental phase to the next implies that the landscape of histone modification is remodelled. Remodelling chromatin marks is the result of combining deposition of new marks and removal of pre-existing marks. Such removal can be achieved by passive dilution upon DNA duplication preceding cell division as shown recently for the control of transcription of gene important in flower development (Sun et al., 2014). In addition, activity of specific enzymes remove histone modifications (Holbert and Marmorstein, 2005; Pedersen and Helin, 2010; Liu et al., 2014) cooperating with the replacement of the entire nucleosome by histone chaperones (Moshkin et al., 2009). In animals, several studies have shown that histone variants are involved in nucleosome replacement leading to removal of the modified histones (Ng and Gurdon, 2008; Hayashi and Surani, 2009; Banaszynski et al., 2013; Lewis et al., 2013). Histone variants are distinct protein isoforms encoded by paralogs of a family of histones genes (Talbert and Henikoff, 2010). In all multicellular eukaryotes four protein families encode the core histone variants H3, H2A, H2B and H4. To the exception of the H4 family, the three other histone families comprise histone variants (Talbert and Henikoff, 2010; Talbert et al., 2012).

Amongst the histone H3 family, the variants H3.1 and H3.3 differ by only a few amino acids. The yeast gene anti-silencing function 1 (asf1) and its human ortholog ASF1A facilitate the assembly the heterodimers of histones H3 and H4 (Park and Luger, 2008). ASF1A interacts with specific complexes Chromatin Assembly Factor 1 (CAF1) and HIRA in the deposition of H3.1 and H3.3, respectively (Corpet and Almouzni, 2009). The variant H3.1 is incorporated in a replication dependent manner by the CAF1 complex that comprises three subunits encoded by p48, p150 and p60 (Verreault et al., 1996), the *Arabidopsis* orthologs of which are *Multicopy Suppressor of IRA1* (*MSI1*), *FASCIATA 1* (*FAS1*) and *FASCIATA2* (*FAS2*), respectively (Kaya et al., 2001; van Nocker, 2003). In contrast with H3.1, the variant H3.3 shows specific features that evolved independently between animals and plants. H3.3 is enriched over gene bodies, predominantly towards the transcription termination site (TTS) and its enrichment level correlates with levels of gene expression (Goldberg et al., 2010; Ray-Gallet et al., 2011; Stroud et al., 2012; Wollmann et al., 2012). RNA polymerase II and H3.3 enrichment profiles overlap over gene bodies (Zlatanova et al., 2007; Ray-Gallet et al., 2011; Wollmann et al., 2012) suggesting that H3.3 participates directly to regulate gene transcription. H3.1 and H3.3 are placed into the chromatin by distinct chaperone complexes (Nakatani et al., 2004; Tagami et al., 2004; Ray-Gallet et al., 2007; Ray-Gallet et al., 2011; Szenker et al., 2011). In mammals and fruit flies, the replacement of H3.3 requires the histone chaperone HIRA (Ray-Gallet et al., 2002; Loppin et al., 2005). HIRA forms a complex with Anti Silencing Factor 1 (ASF1), Calcineurin Binding protein 1 (CABIN1) and Ubinuclein (UBN) 1 and 2 (Tagami et al., 2004; Balaji et al., 2009; Amin et al., 2012). In human HeLa cells, HIRA, UBN1 and ASF1a co-localise to sites enriched in H3.3 in the genome, supporting the function of the HIRA complex in H3.3 deposition (Pchelintsev et al., 2013). CABIN1 interacts through its conserved N-terminal TRP repeats directly with the HIRA C-terminal domain (Rai et al., 2011) while ubinuclein homologs share the conserved domain HRD (Hpc2 related domain) (Balaji et al., 2009) that interacts with the N-terminal WD40 repeats of HIRA (Rai et al., 2011). The loss of function of either HIRA or the UBN1 homolog YEMANUCLEIN in *Drosophila* prevents H3.3 loading on the male pronucleus at fertilization (Loppin et al., 2005; Orsi et al., 2013). This function is likely conserved in other animals (Akiyama et al., 2011). HIRA dependent H3.3 deposition also impacts development in vertebrates (Jullien et al., 2012; Szenker et al., 2012) but its impact is more limited in invertebrates development (Sakai et al., 2009). In plants the HIRA complex has not been isolated. To the exception of HIRA (Phelps-Durr et al., 2005) and ASF1 (Zhu et al., 2011) orthologs of other members have not been identified and the association between HIRA and H3.3 in plants remains unknown. In *Arabidopsis*, H3.3 is dynamically replaced during fertilization in a manner similar to that described in animals (Ingouff et al., 2007; Ingouff et al., 2010) but there has been so far no evidence for specific functions of H3.3 in plant development.

Here we identify the *Arabidopsis* orthologs of genes encoding subunits of the mammalian HIRA complex and provide evidence for their interaction as a complex. We show that HIRA affinity to H3.3 is conserved and that HIRA is active during cell de-differentiation and impacts on pleiotropic aspects of development but is not absolutely required for fertility as in animals. In addition we find a link between HIRA function and pathways involved in responses to environment, suggesting a role of HIRA in plant adaptation.

## MATERIALS AND METHODS

### Plant lines and growth conditions

The wild-type ecotypes Columbia (Col-0), was provided by the Nottingham *Arabidopsis* Stock Centre (NASC). All mutants are in Col-0 background. The mutant alleles *hira-1* (WiscDsLox362H05), *ubn1* (SALK_096291), *ubn2* (SAIL_799_G08), *cabin1* (SALK_106259) were obtained from ABRC at Ohio State University. Seeds were treated at 4°C for 4 days on soil before germination. Plants were grown in growth chamber under short day condition (8-hour day at 20°C/16-hour night at 16°C) for four weeks. Flowering was induced by transferring plants to growth chamber maintained at 22°C with long day condition (16-hour-day and 8-hour-night cycles). Protoplast was generated as previously described (Tessadori et al., 2007).

### Molecular biology and constructs

For each gene encoding a member of the HIRA complex, a genomic fragment was amplified with gene specific attB primers using the KOD-plus-PCR kit (Toyobo). The 3 kb region before the coding region was amplified as a gene specific promoter (supplementary material Table S1). PCR fragments were cloned directionally after a BP reaction (Invitrogen) into the pDONR vector (Invitrogen) to generate entry vectors. Recombination LR reactions were performed between the destination vector and the entry vectors to generate a recombined destination plasmid with an HIRA complex gene with its C-terminal fused to the gene encoding the green fluorescent protein (promoHIRA-HIRA-GFP) or mCherry fluorescent protein (promoHIRA-HIRA-mCherry). These constructs were transformed into wild-type (Col-0 accession) plants by *Agrobacterium tumefaciens*-mediated floral dip method. For each transgene at least 10 T1 lines were selected and used as T2 for further tests. Real-time PCR was done with Power SYBR PCR master mix kits (ABI).

### Transcriptome and bioinformatics analysis

For microarray analysis, Total RNA extracted from protoplast cells and seedling roots using RNeasy Mini kit (Qiagen) hybridized to ATH1 (Affymetrix) array according to the manufacturer's instructions. All CEL files were normalized by gcRMA (Robust Multichip Average) methods (Wu and Irizarry, 2004) and we selected genes with expression levels changed more than 2 times that passed the two-way ANOVA test. H3K27me3 and H2A.Z data were obtained online (Zhang et al., 2009; Coleman-Derr and Zilberman, 2012). Gene Ontology analysis was performed on the DAVID web server (http://david.abcc.ncifcrf.gov). Phylogenetic analysis was performed online (http://www.phylogeny.fr).

### Immunofluorescence

Immunofluorescence staining and microscopic observations were performed as previously described (Jacob et al., 2009). Different fluorescence signals were imaged by using a laser scanning confocal microscope (Zeiss LSM 510 META). Images were processed with Illustrator CS3 and Photoshop CS3 (Adobe). Co-localisation analysis was performed using Imaris (Bitplane).

### Coimmunoprecipitation

Coimmunoprecipitation experiments were performed as previously described (Fiil et al., 2008). Ten-day-old seedlings or four-week-old rosette leaves were used for nuclei extraction. Tagged proteins were pulled down using tag-specific antibodies and detected in western blot. Anti-GFP (ab290, Abcam), anti-mCherry (ab167453, Abcam) and anti-H3 (ab1791, Abcam) antibodies were used.

## RESULTS

### HIRA affects vegetative but not reproductive development

**Fig. 1.**
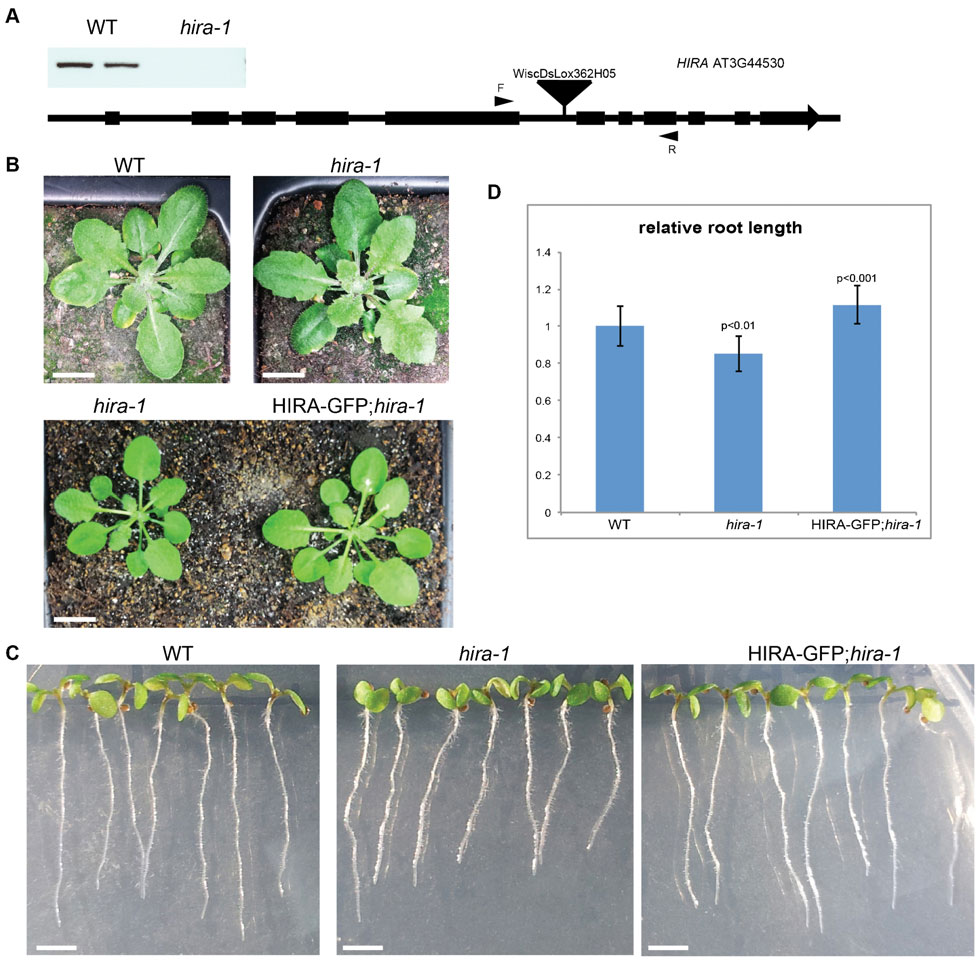
*hira* shows mild pleiotropic developmental defect. (A) Schematics drawing of T-DNA insertion map. The black triangle marks the position of T-DNA insertion line WiscDxLox_362H05. Using primers marked by black arrowheads, no *HIRA* transcript is detected in *hira-1*. (B) Vegetative phenotypes of a *hira-1* plant with accentuated leaf serration compared to a wild-type plant. The leaf phenotype is rescued by HIRA-GFP. Scale bars: 1 cm. (C) Five-day-old seedlings of WT, *hira-1* and HIRA-GFP;*hira-1*. Root length of *hira-1* is significantly shorter than WT and cotyledons are bent downward. Scale bars: 2 mm. (D) Relative root length measurement of WT, *hira-1*, and HIRA-GFP;*hira-1*.

The *Arabidopsis* genome contains a single ortholog (At3g44530, *HIRA*) of the human gene *HIRA*. We observed the conservation of the functional domains of the predicted HIRA protein as defined in metazoans (supplementary material Fig. S1A). A phylogenetic comparison of HIRA orthologs suggests that HIRA evolved from a single ancestral gene in multicellular eukaryotes independently in plants, fungi and animals and that only a single gene is conserved in each genome (supplementary material Fig. S1B). Amongst lines with a T-DNA insertion in the gene body of *HIRA* in *Arabidopsis* we selected the line WiscDsLox_362H05 with a T-DNA inserted in the fifth intron of *HIRA*. In this line the full *HIRA* transcript was not detected (Fig. 1A) and we concluded that this line is a null mutant allele that we named *hira-1*. We analysed the transmission of *hira-1* and found that self-fertilized *hira-1*/+ plants produced 25% homozygous *hira-1* viable plants and that backcrosses between WT and *hira-1*/+ plants produced 50% of *hira-1*/+ plants (Table 1) indicating that the loss of function of HIRA does not affect viability of gametes and embryos. As expected, flowers from homozygous *hira-1* plants were similar to WT and detailed observation of developing siliques showed that in self-pollinated *hira-1/hira-1* plants all ovules were fertilized, producing viable seeds similar to WT (supplementary material Fig. S1C). Similarly, we could not identify embryo arrest in siliques produced from plants homozygous for the *hira-1* allele described previously, which contains a T-DNA inserted in the 5′ untranslated region of *HIRA* (Phelps-Durr et al., 2005) (supplementary material Fig. S1D). We detected levels of *HIRA* transcripts similar to WT levels in this allele (supplementary material Fig. S1D), which suggested that HIRA function was not compromised in this line. We thus focused our studies on the allele *hira-1*. Plants homozygous for *hira-1* were fertile and showed slight pleiotropic defects in vegetative development. Rosettes of adult *hira-1* plants showed leaves with higher degree of serration than in wild types (Fig. 1B). In *hira-1* seedlings, root growth was slightly retarded and five days after germination, cotyledons were bent downward in contrast to WT (Fig. 1C,D). We obtained *hira-1/hira-1* plants expressing the protein HIRA fused to the GREEN FLUORESCENT PROTEIN (HIRA-GFP) under the control of the endogenous *HIRA* promoter. These transgenic plants resembled wild-type plants and did not display the vegetative phenotypes of *hira-1* mutants (Fig. 1B–D). We concluded that HIRA-GFP complemented the null allele *hira-1* and that the vegetative phenotypes observed in *hira-1* were caused by the loss of function of HIRA.

**Table 1.**
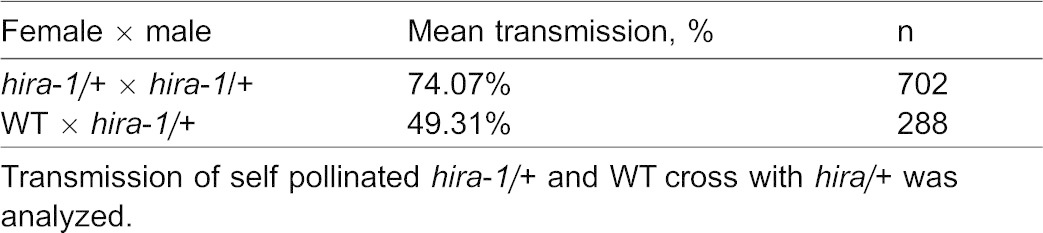
Genetic analysis of *hira-1* transmission

### The *Arabidopsis* HIRA complex

We searched *Arabidopsis* orthologs of all members of the HIRA complex described in mammals. In the *Arabidopsis* genome we found a high degree of sequence homology with human UBINUCLEIN 1 and 2 in the genes AT1G21610 and AT1G77310 that we named *AtUBN1* and *AtUBN2*, respectively (supplementary material Fig. S2A). Protein alignments showed that orthologs of UBN1 share three domains that are conserved between plants, yeasts and metazoan (supplementary material Fig. S2B,C). The phylogeny of *UBN1* family suggests that *UBN1* evolved separately in the plant lineage with a tendency for gene duplication and preservation of paralogs (supplementary material Fig. S2D). As a potential ortholog of the human CABIN1 (Calcineurin Binding protein 1) we identified the gene AT4G32820 that we named *AtCABIN1* (supplementary material Fig. S2E). The TPR domain of CABIN1 is conserved to a significant degree between metazoan and plants (supplementary material Fig. S2F).

From the expression profiles available online we observed that the expression profiles of *UBN1*, *UBN2*, *CABIN1* and *HIRA* are cell cycle independent in contrast with the expression profiles of *FAS1*, and *FAS2* that encode the CAF1 specific subunits (supplementary material Table S2). These genes are expressed at similar levels in all vegetative tissues during plant development and at levels lower than the genes encoding most other members of the CAF1 complex (supplementary material Fig. S3A).

**Fig. 2.**
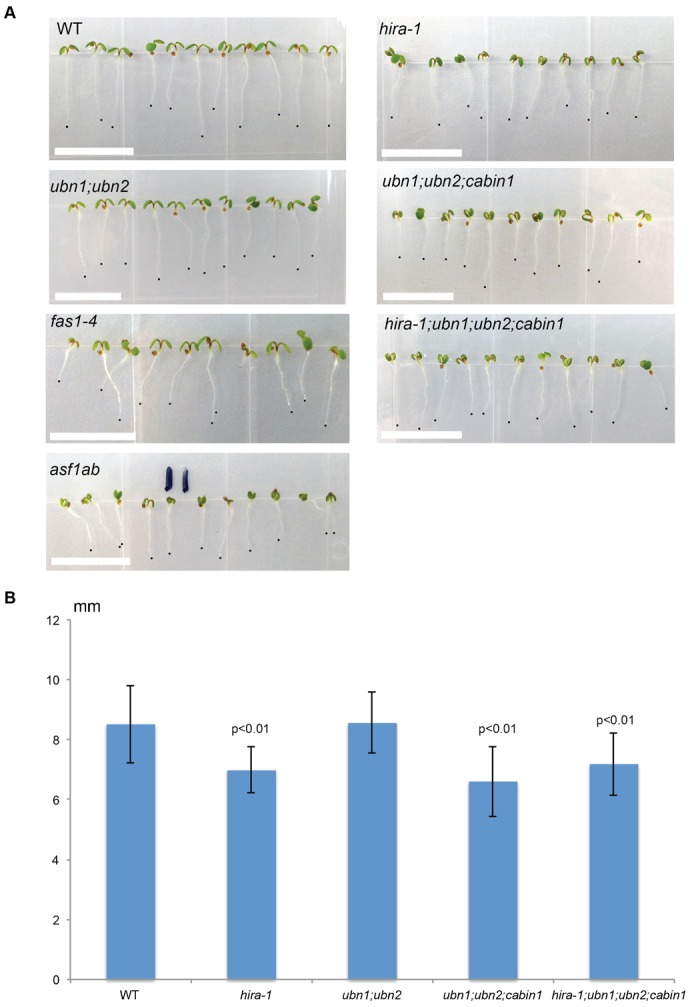
Subunits of the HIRA complex interact in the same genetic pathway. (A) Five-day-old seedlings of WT, *hira-1*, *ubn1;ubn2*, *ubn1;ubn2;cabin1*, *fas1-4*, *hira-1;ubn1;ubn2;cabin1* and *asf1ab*. Black dots mark the root tip. Scale bars: 1 cm. (B) Root length measurements of WT, *hira-1*, *ubn1;ubn2*, *ubn1;ubn2;cabin1* and *hira-1;ubn1;ubn2;cabin1*.

We isolated the null mutant alleles *ubn1*, *ubn2* and *cabin1* (supplementary material Fig. S3B,C) but none of the single or double homozygous mutants showed developmental defects as shown by *hira-1* (Fig. 2A and not shown). However, five-day-old seedlings of the triple homozygous mutant *ubn1;ubn2;cabin1* and the quadruple homozygous mutant *hira-1;ubn1;ubn2;cabin1* were fertile (supplementary material Fig. S3D) and showed a vegetative phenotype similar to *hira-1* including reduced root growth and bent-cotyledons phenotype (Fig. 2A,B), supporting that these genes participate in the same genetic pathway. The bent-cotyledons phenotype was also observed in the double homozygous mutant *asf1a;asf1b*, supporting that in *Arabidopsis* HIRA, CABIN1, UBN1, UBN2, ASF1a and ASF1b act in the same pathway. The absence of bent-cotyledons in *fas1* seedlings suggests that ASF1 function in the HIRA pathway is independent from its function in the CAF1 complex activity.

### HIRA interacts with H3.3

**Fig. 3.**
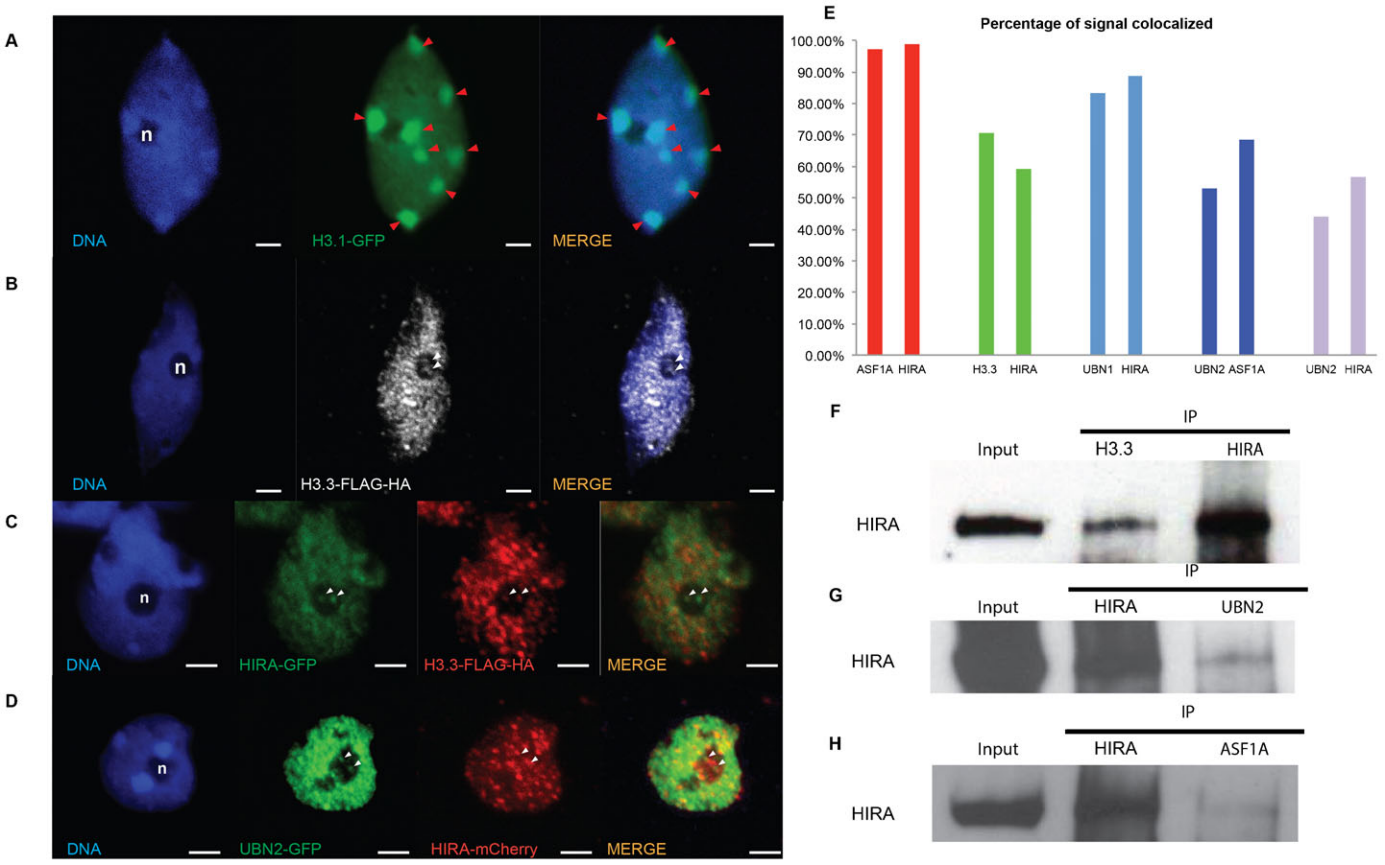
HIRA complex subunits interact with each other. Immunolocalisation of (A) H3.1, (B) H3.3, (C) HIRA and H3.3, (D) UBN2 and HIRA. Note the similar localisation of HIRA and UBN2 with H3.3 but not H3.1, especially inside the nucleolus. Scale bars: 2 µm. (E) Percentage of signal co-localised in nucleus of different subunits of HIRA complex. Coimmunoprecipitation shows that HIRA interact with (F) H3.3, (G) UBN2 and (H) ASF1A. Red arrowheads mark chromocenters, white arrowheads mark rDNA loci, and “n” marks nucleolus.

In order to establish whether HIRA associates preferentially with H3.3 over H3.1 we first studied the localisation of HIRA. Using stable transgenic lines that express H3.1-GFP (Fig. 3A) we confirmed that H3.1 labels both euchromatin and constitutive heterochromatin that is represented by condensed regions of chromatin marked by DAPI called chromocenters (Ingouff et al., 2010; Wollmann et al., 2012). In contrast, immunodetection of FLAG tag in stable transgenic lines that express H3.3-FLAG-HA showed that H3.3 marks dots in the nucleolus (Fig. 3B). Such dots correspond to the actively transcribed genes encoding ribosomal RNAs as reported previously (Shi et al., 2011). In a transgenic plant line that co-expresses H3.3-FLAG-HA and HIRA-GFP we observed that histone H3.3-FLAG-HA co-localised with HIRA-GFP in euchromatin, including actively transcribed genes encoding ribosomal RNAs (Fig. 3C). The colocalisation of HIRA and H3.3 in euchromatin is compatible with a role of HIRA in H3.3 replacement at active genes.

In order to test the interaction between HIRA and H3.3 further, we used a stable transgenic plant line that co-expresses H3.3-GFP and 35S-HIRA-mCherry. We detected HIRA-mCherry in the chromatin pull-down by an antibody against H3.3-GFP (Fig. 3F), supporting further that HIRA binds to H3.3. To test whether HIRA forms a complex similar to that described in mammals we obtained transgenic plant lines that express UBN2 fused to mCherry and HIRA-GFP. These two proteins showed a very similar pattern of localisation inside the nucleus (supplementary material Fig. S4). Co-expressed UBN2-GFP and HIRA-mCherry shown high level of co-localisation inside nucleus (Fig. 3D,E). In addition, pull-down experiments showed that HIRA and UBN2 interact (Fig. 3G). Using a similar strategy we also observed the co-localisation and interaction between HIRA, UBN1 and ASF1A. (Fig. 3E,H). Together with the genetic analyses these results support that in *Arabidopsis* HIRA associates with UBN1/UBN2, CABIN1 and ASF1 to perform its function as a complex comparable to the HIRA complex reported in mammals (Tagami et al., 2004; Banumathy et al., 2009).

### A pleiotropic function of HIRA on development and reprogramming

**Fig. 4.**
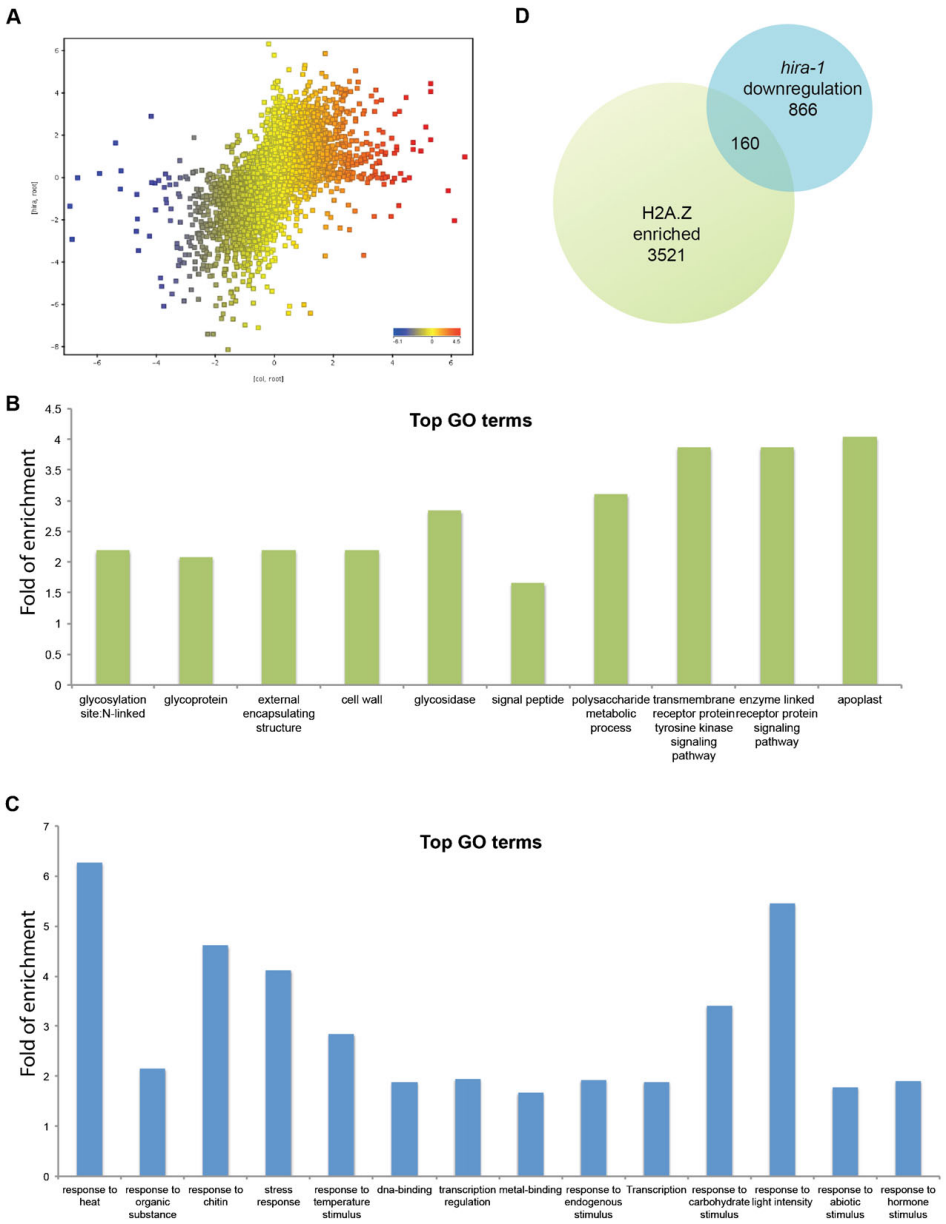
HIRA affects expression of responsive genes. (A) Scatter plot of transcriptome comparison between WT and *hira-1* root cells. Top GO terms from (B) up-regulated and (C) down-regulated genes in *hira-1* compared with WT. (D) Venn diagram of down-regulated genes in *hira-1* and H2A.Z enriched genes.

To assess the impact of HIRA on development we chose roots the growth of which is retarded in *hira-1*. A comparison between the transcript profiles of wild-type and *hira-1* roots from seven-day-old seedlings showed that 1095 genes were more expressed and 1026 genes were less expressed in *hira-1* mutant than in WT, when a threshold of more or less than twofold change was applied (Fig. 4A). Most of the genes up-regulated did not define a specific type of function, outlining either indirect effects or a pleiotropic function of HIRA (Fig. 4B). By contrast, GO terms corresponding to genes down-regulated in *hira-1* played a role in responses to biotic and abiotic environment (Fig. 4C) and belonged to the broad class of responsive genes defined previously (Aceituno et al., 2008; Coleman-Derr and Zilberman, 2012). The expression of responsive genes is up-regulated in plants that are deprived of the histone variant H2A.Z (Coleman-Derr and Zilberman, 2012). In contrast, the expression of responsive genes was down-regulated in *hira* mutant and we observed that a significant proportion of these genes tend to be also enriched in H2A.Z in WT background (Fig. 4D), suggesting a functional link between H2A.Z enrichment and the function of HIRA, although this link might be indirect.

**Fig. 5.**
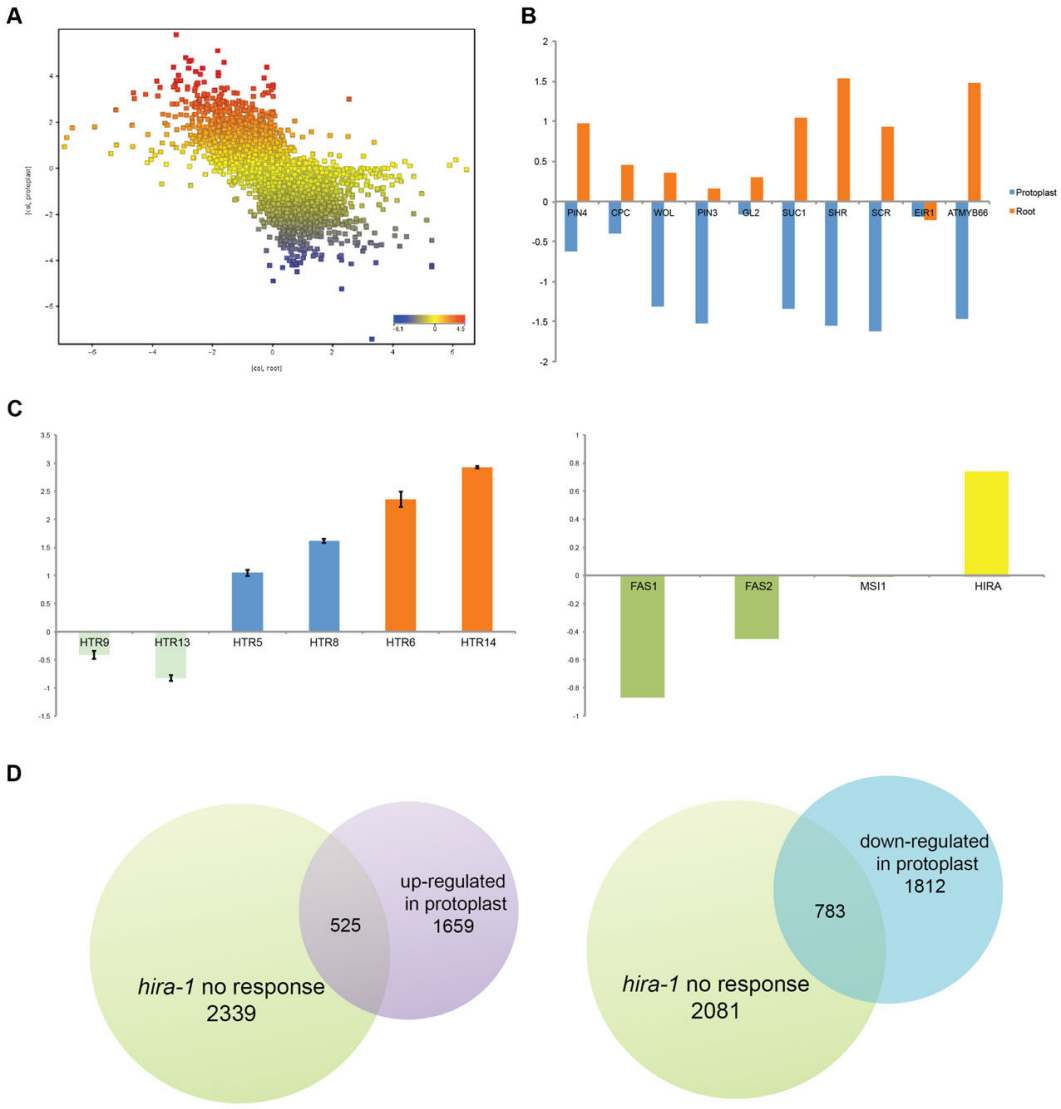
HIRA affects dedifferentiation. (A) Transcriptome comparison between WT root cells and WT protoplast cells. (B) Specific root markers down-regulated in protoplasts. (C) Histone variants and chaperones show different transcriptional profiles between WT root cells and WT protoplasts. (D) Venn diagrams comparing genes that are not affected by *hira-1* mutation with genes that are down- or up-regulated in protoplasts versus root cells.

Plant development is characterized by the capacity to reprogram differentiated cells to initiate asexual reproduction and to produce new organs such as lateral roots. To test whether HIRA could be involved in aspects of developmental reprogramming we studied the effect of HIRA during de-differentiation. We removed the extra cellular matrix of root cells to produce protoplasts, which are able to initiate a process leading to plant regeneration. Protoplasts obtained from roots were characterized by a deeply modified transcriptome (Fig. 5A). 21% of genes were differentially expressed in protoplasts relative to wild-type roots that were not treated with enzymatic digestion of the cell wall. These genes included markers of root cell types (Fig. 5B) indicating the loss of differentiated state typical of protoplasts. Protoplasts also experienced an arrest of cell cycle that was marked by down-regulation of cell cycle regulators (supplementary material Fig. S4A). Genes encoding the S-phase dependent histone variant H3.1 and subunits FAS1 and FAS2 of the CAF1 complex that deposits H3.1 were also down-regulated (Fig. 5C). In contrast, the expression levels of genes encoding the variant H3.3 increased significantly together with the expression of HIRA (Fig. 5C). The effect of the enzymatic treatment leading to protoplast production was effective within 8 h (supplementary material Fig. S5B), a duration that is smaller than the average duration of a cell cycle in *Arabidopsis*. From these observations, we hypothesized that dedifferentiation of protoplasts involved DNA replication independent incorporation of H3.3 by HIRA causing a dramatic change of transcriptome profile in protoplasts.

We compared the transcriptome profile of protoplasts obtained from *hira-1* mutant roots with the profile of protoplasts from wild-type roots used in the experiment reported in Fig. 5A. Protoplasts from *hira-1* root also underwent a significant change in transcript profiles compared to untreated roots. However, *hira* protoplasts did not show changes of transcript levels in 24% and 30% of the genes that were up-regulated and down-regulated in wild-type protoplasts, respectively (Fig. 5D). This requirement of HIRA in a significant proportion of the gene affected by a transcriptional change in dedifferentiated protoplasts suggests that HIRA participates in dedifferentiation of plant cells.

## DISCUSSION

### Molecular conservation of the HIRA complex in plants

We identified *Arabidopsis* orthologs of all genes encoding partners of the HIRA complex described in mammals comprising HIRA, UBN1 and CABIN1. The conservation of the NHRD domain amongst *Arabidopsis UBN1* and *UBN2* paralogs suggests the conservation of its function in interacting with HIRA WD40 repeats (Tang et al., 2012) is conserved between mammals and plants. Overall, plants orthologs of HIRA, UBN1 and CABIN1 share conserved domains showing a fairly high degree of homology with the domains described for each partner of the HIRA complex in mammals. This suggests conservation of a putative HIRA complex throughout the kingdom Plantae.

The genome of extant angiosperm *Amborella*, that separated from other angiosperms before the polyploidization gamma (Soltis et al., 2008), contains a single ortholog of *UBN1* while two orthologs are present in genomes of other angiosperms including *Arabidopsis* and rice. Duplication of a plant ancestral *UBN1* ortholog took place in ferns and mosses as well, suggesting that *UBN1* paralogs acquired specialized functions several times during plant evolution. However, so far in *Arabidopsis*, the expression patterns of *UBN1* and *UBN2* are similar and genetic data rather support functional redundancy. Similarly, duplication of the *ASF1* ortholog took place and was maintained in plants and in mammals. In mammals ASF1a and ASF1b participate to distinct complexes involved in H3 variant deposition (Abascal et al., 2013). In *Arabidopsis*, the distinct expression profiles of *ASF1A* and *ASF1B* also suggest distinct functions for these two paralogs.

The similar phenotype observed in seedlings from *hira*, the triple mutant *ubn1;ubn2;cabin1*, and the quadruple mutant *hira-1;ubn1;ubn2;cabin1* indicate that these four genes interact in the same genetic pathway. The absence of phenotype in single mutants for UBN1, UBN2 and CABIN1 hints that these subunits are not essential on their own to HIRA function and show a degree of redundancy. The co-localisation between HIRA, UBN1, UBN2 and ASF1 and the evidence supporting binding between HIRA and UBN2 or ASF1 altogether support that HIRA, UBN1 or UBN2, ASF1A or ASF1B and CABIN1 form together a complex similar to the HIRA complex identified in mammals.

In mammals and fruit fly, the HIRA complex plays a specific function in the deposition of H3.3 variants. In *Arabidopsis* HIRA and UBN1 co-localise with H3.3 in euchromatin but the degree of co-localisation with H3.1 remains unclear. Pull-down experiments support that H3.3 and HIRA interact. However, a firm conclusion regarding the specificity of the *Arabidopsis* HIRA complex towards H3.3 versus H3.1 awaits further *in vitro* reconstitution of the HIRA complex.

### HIRA is not required for plant fertilization

In animals, loss of function of HIRA affects severely fertilization and early embryogenesis. In *Drosophila*, HIRA is essential for the replication-independent incorporation of H3.3 in the male pronucleus and its absence prevents male genome decondensation at the first mitosis leading to male genome elimination (Loppin et al., 2005). A similar phenotype was described in *Drosophila* deficient in *Yemanuclein*, the ortholog of *UBN1* (Orsi et al., 2013). Similar to mammals (Akiyama et al., 2011), *Arabidopsis* H3.3 is expressed immediately after fertilization and replaces the unusual H3.10 variant present in male gamete nuclei (Ingouff et al., 2010). Although this similarity suggests that HIRA is important in zygote chromatin dynamics in *Arabidopsis*, null alleles of *hira*, *ubn1*, *ubn2* and *cabin1* and their combinations are fertile and do not show defects in fertilization or early embryogenesis. This lack of impact of HIRA is likely explained by redundancy originating either from another H3.3 incorporation pathway depending on *Arabidopsis* orthologs of DAXX or DEK that encode H3.3 chaperones in mammals (Sawatsubashi et al., 2010; Elsässer et al., 2012).

### HIRA involvement in dedifferentiation and chromatin dynamics

We observe that HIRA participates in the remodelling that accompanies dedifferentiation of protoplasts within a few hours, a time shorter than required for one cell cycle. This supports that HIRA participates to incorporation of the replication independent H3.3 rather than H3.1 during dedifferentiation of protoplasts. Such a role is similar to that described in vertebrates where HIRA is involved in reprogramming transcription following nuclear transfer in *Xenopus* (Jullien et al., 2012) and in the differentiation of cell lines (Song et al., 2012).

In the plant root, the loss of HIRA function affects responsive genes. These genes have been defined by a large number of transcriptome studies available for different tissue types or responses to various environmental or hormonal stimuli (Aceituno et al., 2008; Coleman-Derr and Zilberman, 2012). Interestingly there is a tight link between gene body enrichment of the histone variant H2A.Z and gene responsiveness (Coleman-Derr and Zilberman, 2012) including response to temperature (Kumar and Wigge, 2010) and we also found that HIRA dependent responsive genes are enriched in H2A.Z. H2A.Z is predominantly enriched over 5′ gene ends, at gene promoters/transcription start sites (TSS) (Zilberman et al., 2008). This pattern is symmetrical with H3.3 enrichment toward the 3′ end of genes and H2A.Z and H3.3 enrichments are anti-correlated (Stroud et al., 2012). It is thus possible that incorporation of H3.3 by HIRA plays a role in the confinement of H2A.Z to the 5′ end of genes. The effect of the loss of HIRA on responsive genes might thus reflect a broader property of the role of HIRA in H3.3 dynamics that are linked with the dynamics of transcription that accompany developmental transitions and responses to the environment.

In conclusion, our data suggest that the function of HIRA complex as a histone H3.3 chaperone is conserved in *Arabidopsis*. The high degree of conservation between plants and animals indicate the importance of this protein complex during evolution. It is not surprising that although pleiotropic, the defects are limited in *hira* mutants similar to that observed in *caf1* and *asf1* mutants in *Arabidopsis* (Kaya et al., 2001; Zhu et al., 2011). Yet, our data reveal that HIRA plays important roles during reprogramming events associated with differentiation and responsiveness to the environment. Whereas a role of HIRA in development is likely shared between plants and animals, it is possible that sessile plants have enhanced the importance of HIRA replication independent chromatin dynamics to cope with daily changing abiotic conditions and adapt to seasonal variations.

## Supplementary Material

Supplementary Material
