# Supplementary material for: The HIRA complex that deposits the histone H3.3 is conserved in Arabidopsis and facilitates transcriptional dynamics
Source: Biol Open. 2014 Aug 1;3(9):794–802. doi: 10.1242/bio.20148680 (PMC4163656; doi:10.1242/bio.20148680)
Supplement: Supplementary Material [file supp_3_9_794__index.html]

The HIRA complex that deposits the histone H3.3 is conserved in Arabidopsis and facilitates transcriptional dynamics — Supplementary Material 

# The HIRA complex that deposits the histone H3.3 is conserved in *Arabidopsis* and facilitates transcriptional dynamics

## bio.20148680 Supplementary Material

**Files in this Data Supplement:**

- Supplementary Material - Xin Nie et al. doi: 10.1242/bio.20148680
