# Supplementary material for: The HIRA complex that deposits the histone H3.3 is conserved in Arabidopsis and facilitates transcriptional dynamics
Source: Biol Open. 2014 Aug 1;3(9):794–802. doi: 10.1242/bio.20148680 (PMC4163656; doi:10.1242/bio.20148680)
Supplement: Supplementary Material [file supp_bio.20148680_bio.20148680-s1.pdf]

Biology Open (2014) 000, 1–9 doi:10.1242/bio.20148680

Supplementary Material
Xin Nie et al. doi: 10.1242/bio.20148680

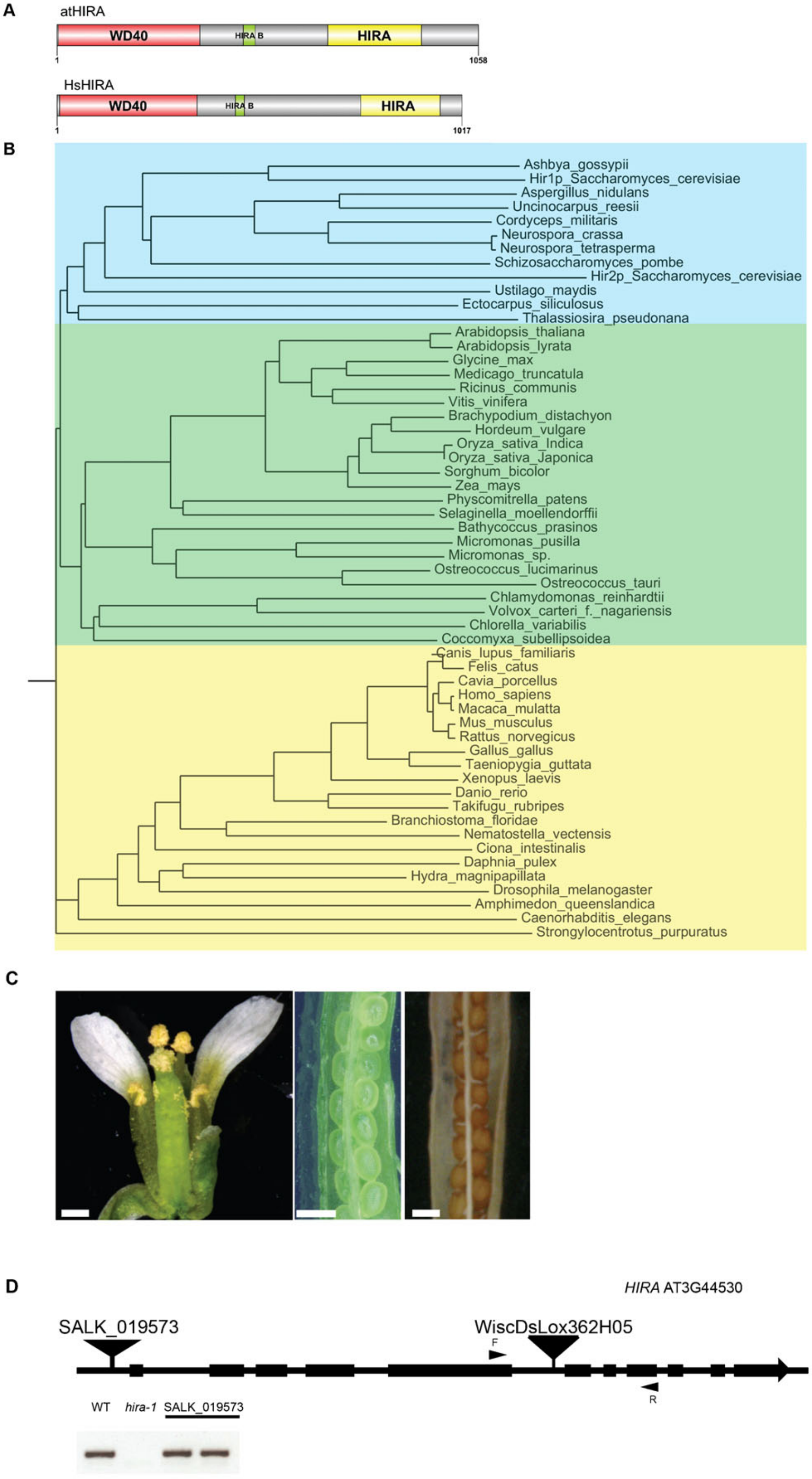

Fig. S1. HIRA is conserved in *Arabidopsis*. (A) Functional domain comparison between *Arabidopsis* (AtHIRA) and human (HsHIRA). (B) Phylogenetic analysis of HIRA in different organisms. Each kingdom is marked by a distinct color. (C) Representative picture of *hira* flower organ and silique. (D) Schematic drawing of the T-DNA insertion map of the allele SALK 019573. The gel shows the presence of HIRA transcripts in contrast to the *hira-1* allele. Scale bars: 0.5 mm.

Biology Open

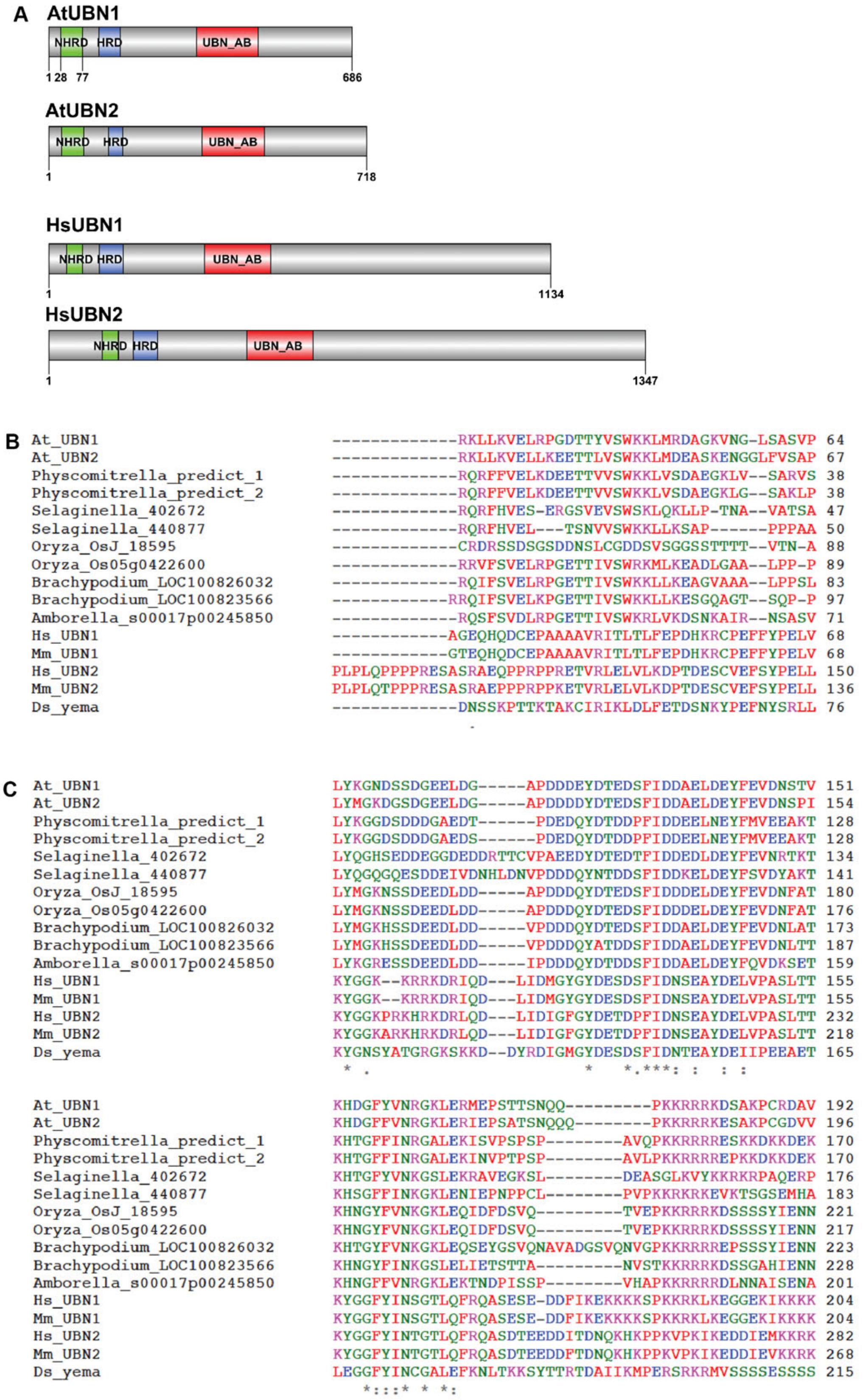

continued

Fig. S2. The HIRA complex subunits in *Arabidopsis*. (A) Functional domain alignment of AtUBN1, AtUBN2, HsUBN1 and H2UBN2. (B) N-terminal alignment of UBN1 orthologs. (C) HRD domain alignment of UBN1 orthologs. (D) Phylogenetic analysis of UBN1 orthologs. (E) Functional domain alignment of AtCABIN1 and HsCABIN1. (F) TPR domain alignment of AtCABIN1, MmCABIN1 and HsCABIN1.

Biology Open

 Biology Open (2014) 000, 1–9 doi:10.1242/bio.20148680

Fig. S2. continued

D

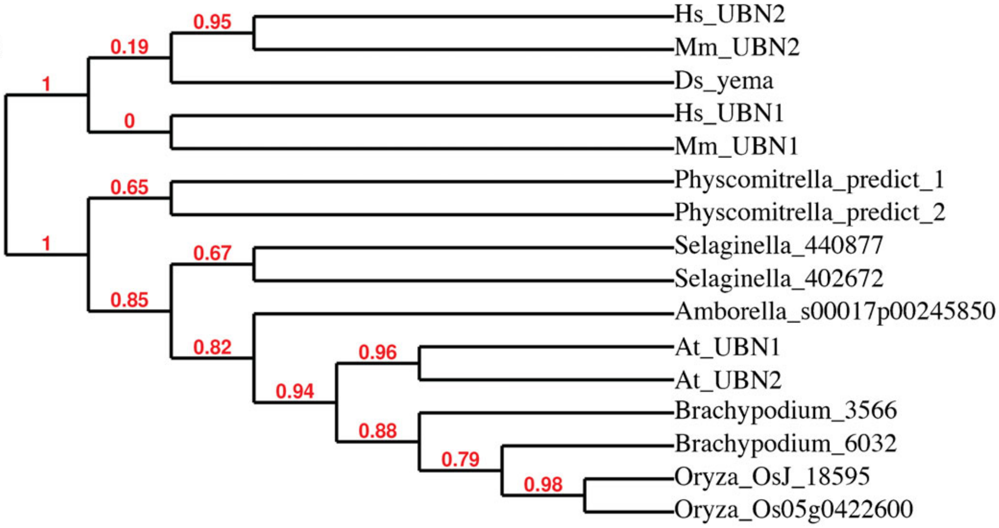

E

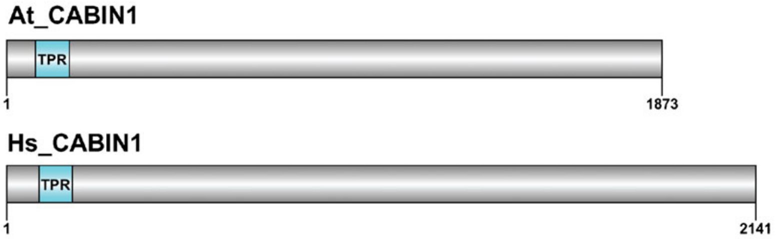

F

```
Mm_CABIN1       MIRIAALNASSTIEDDHEGSFKSHKIQTKEAQEAEAFALYHKALDLQKHDRFEESAKAYH 60
Hs_CABIN1       MIRIAALNASSTIEDDHEGSFKSHKTQTKEAQEAEAFALYHKALDLQKHDRFEESAKAYH 60
At_CABIN1       MFSIAAINDTESTEKWEP------LAPSKEAQEFHLSQTYHDGLLKLQAKDYDKARELLE 54
                *: ***:* :.: *. .          :***** .    **..*   : . :::: :  .

Mm_CABIN1       ELLEARLLREAVSSGDEKEGLKHPGLILKYSTYKNLAQLAAQR--EDLETAMEFYLEAVM 118
Hs_CABIN1       ELLEASLLREAVSSGDEKEGLKHPGLILKYSTYKNLAQLAAQR--EDLETAMEFYLEAVM 118
At_CABIN1       SILKDPIITNSKVETIANDNHLHH---LRFLALKNLATVFLELGSSHYENALNCYLQAID 111
                .:*:  :: ::  .   ::.  *    *:: : **** :  :   .. *.*:: **:*:

Mm_CABIN1       LDSTDVNLWYKIGHVALRLIRLPLARHAFEEGLRCNPDHWPCLDNLITVLYTLSDYTTCL 178
Hs_CABIN1       LDSTDVNLWYKIGHVALRLIRIPLARHAFEEGLRCNPDHWPCLDNLITVLYTLSDYTTCL 178
At_CABIN1       LDAKDSVLWNHLGTLSCSMGLLSISRWAFEQGLLCSPNNWNCMEKLLEVLIAVGDEVSCL 171
                **:.*  ** ::* ::  :  :.::* ***:** *.*::* *:::*: ** ::.* .:**

Mm_CABIN1       YFICKALEKDCRYSKGLVLKEKIFEEQPCLRKDSLRMFLKCDMSIHDVSVSAAETQAIVD 238
Hs_CABIN1       YFICKALEKDCRYSKGLVLKEKIFEEQPCLRKDSLRMFLKC------------------- 219
At_CABIN1       SVANLILRHWPSHSRALHVKHCIEDTDSAPFAPKGIDKLEP------------------- 212
                 .    *.:   :*:.* :*. * : :..    .    *:
```


Biology Open

Biology Open (2014) 000, 1–9 doi:10.1242/bio.20148680

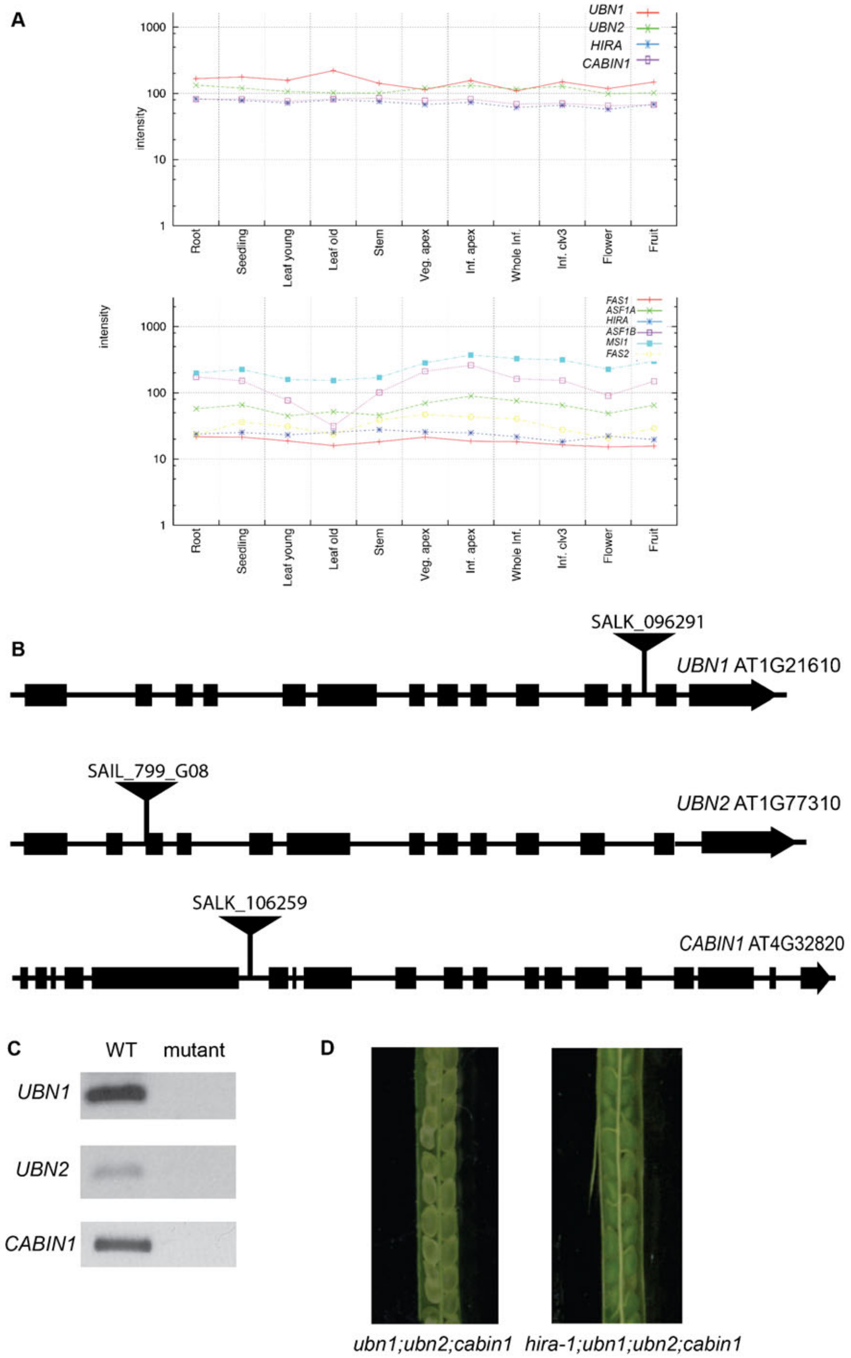

Fig. S3. Expression profiles and mutant alleles of genes encoding subunit of the HIRA complex in *Arabidopsis*. (A) Expression profiles of HIRA complex genes, *CAF1* and *ASF1* through different developmental stages. (B) Schematic drawing of gene loci and T-DNA insertion map of *AtUBN1*, *AtUBN2* and *AtCABIN1*. (C) RT-PCR gel shows the absence of transcript in *ubn1*, *ubn2*, *cabin1*. (D) Silique pictures of *ubn1;ubn2;cabin1* and *hira-1;ubn1;ubn2;cabin1* plants.

Biology Open (2014) 000, 1–9 doi:10.1242/bio.20148680

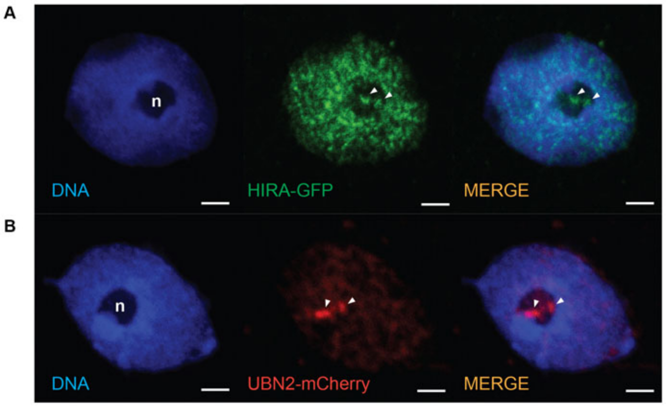

Fig. S4. Immunostaining of HIRA complex in somatic cell nucleus. Immunostaining of (A) HIRA-GFP and (B) UBN2-mCherry. White arrowheads mark rDNA loci, and ''n'' marks nucleolus. Scale bars: 2 μm.

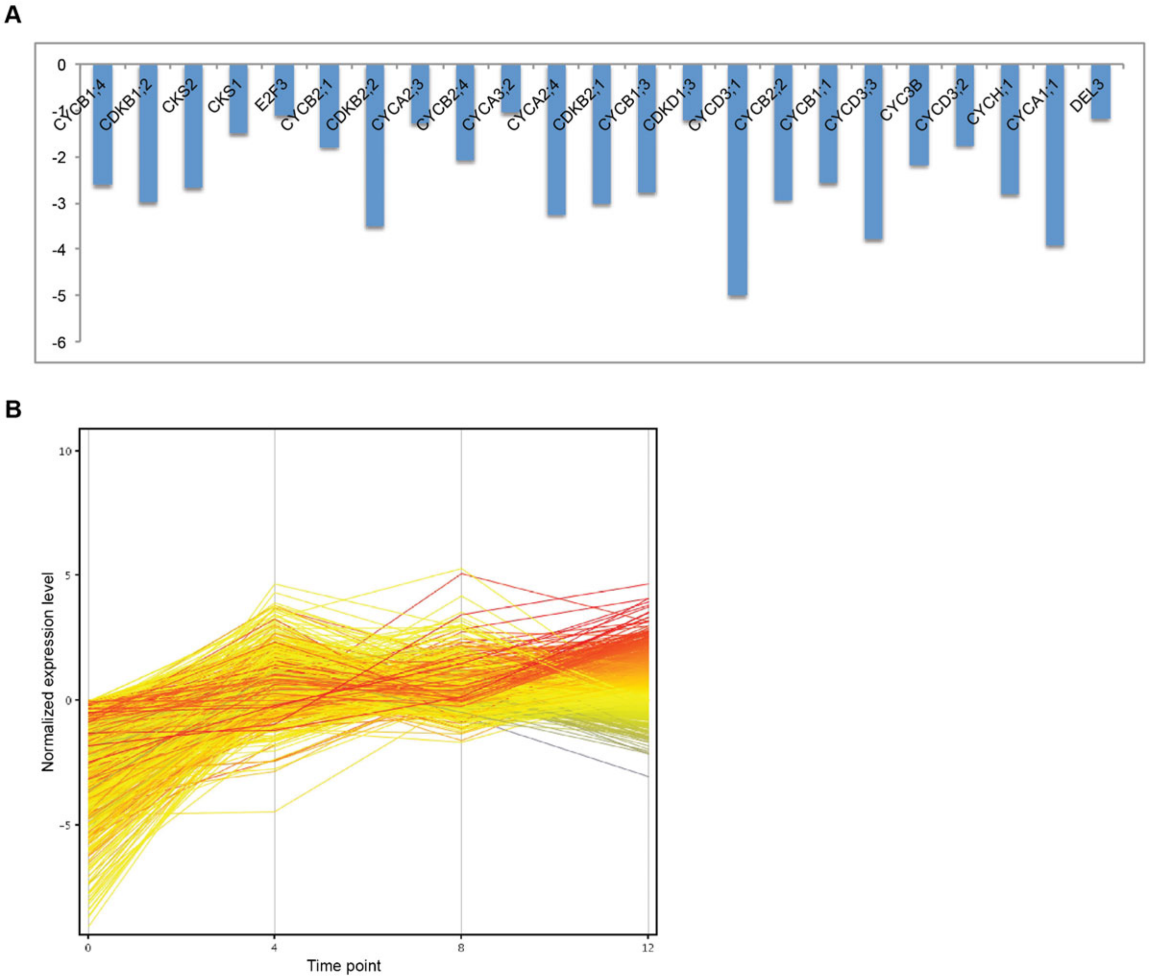

Fig. S5. Dedifferentiation process of protoplasts. (A) Expression of cell cycle regulators is down-regulated in WT protoplasts compared with WT root cells. (B) Transcriptome profile during the production of protoplasts at 4 h, 8 h and 12 h after initiation of enzymatic digestion.

Biology Open (2014) 000, 1–9 doi:10.1242/bio.20148680

Table S1. Primers list

HIRA FORWARD	GAGAGTCACTGTTTTGGCTGG
HIRA REVERSE	CTACTAAAATTTGAGGCCGGG
UBN1 FORWARD	TGGAATCACAGGCAATCAAA
UBN1 REVERSE	TAGTGACCACGGAAGTGCTG
UBN2 FORWARD	GGATGAACATTCAGGTTCACAA
UBN2 REVERSE	TATGTGCCTTGGACTGTGGA
CABIN1 FORWARD	GCCAGAAAGTTCACGGAAA
CABIN1 REVERSE	CTGGCCATATGCTTCCTTGT
ATTB1-HIRA-F	GGGGACAAGTTTGTACAAAAAAGCAGGCTGTGTCTTCCAAATTTGTCACATTTAGCC
ATTB2-HIRA-R	GGGGACCACTTTGTACAAGAAAGCTGGGTGAGAGCCCGAGTCTCTTGAGTTC
ATTB1-UBN1-F	GGGGACAAGTTTGTACAAAAAAGCAGGCTGTTTTTGGTATCCCGCGCA
ATTB2-UBN1-R	GGGGACCACTTTGTACAAGAAAGCTGGGTGACTTGGCAAGTTCAGGTCCG
ATTB1-UBN2-F	GGGGACAAGTTTGTACAAAAAAGCAGGCTGTCCCGCGCAACGTTGTAAC
ATTB2-UBN2-R	GGGGACCACTTTGTACAAGAAAGCTGGGTGACTTGGCAGGTTCAGGTCTGG
ATTB1-ASF1A-F	GGGGACAAGTTTGTACAAAAAAGCAGGCTGTCATTTTCATCGTCTTCTTAATCAAAA
ATTB2-ASF1A-R	GGGGACCACTTTGTACAAGAAAGCTGGGTGTGATTCCTCAGGTTTTGGTTCTAC

Table S2. Cell cycle dependence of different chaperones

Gene name	Rank	Peaktime	P(per)	P(reg)
FAS1	340	S	0.002	0.3
FAS2	2243	uncertain	0.0188	1.726
MSI1	6931	uncertain	0.1115	1.9609
HIRA	13620	uncertain	0.536	2.194
UBN1	12934	uncertain	0.508	1.904
UBN2	19571	uncertain	1.966	2.343
CABIN1	NA	NA	NA	NA
ASF1A	10888	uncertain	0.257	2.304
ASF1B	15230	uncertain	1.362	1.147

Expression profile of different chaperones during cell-cycle. P(per) stand for p-value for Periodicity. P(reg) stand for p-value for regulation.
